# The contribution of agricultural insecticide use to increasing insecticide resistance in African malaria vectors

**DOI:** 10.1186/s12936-016-1162-4

**Published:** 2016-02-19

**Authors:** Molly C. Reid, F. Ellis McKenzie

**Affiliations:** Maryland Institute of Applied Environmental Health, University of Maryland School of Public Health, 22242 Valley Drive, College Park, MD 20742 USA; Fogarty International Center, National Institutes of Health, Bethesda, MD USA

## Abstract

The fight against malaria is increasingly threatened by failures in vector control due to growing insecticide resistance. This review examines the recent primary research that addresses the putative relationship between agricultural insecticide use and trends in insecticide resistance. To do so, descriptive evidence offered by the new research was categorized, and additional factors that impact the relationship between agricultural insecticide use and observed insecticide resistance in malaria vectors were identified. In 23 of the 25 relevant recent publications from across Africa, higher resistance in mosquito populations was associated with agricultural insecticide use. This association appears to be affected by crop type, farm pest management strategy and urban development.

## Background

Malaria is among the most devastating infections in human history. The World Health Organization (WHO) reported 584,000 deaths due to malaria in 2014, 90 % of which were from sub-Saharan Africa [1]. The single most effective tool to combat malaria is vector control, which addresses the source of transmission at a low overall cost. Vector control for malaria is mainly composed of insecticide-treated net (ITN) and indoor residual spraying (IRS) programmes, which rely on a limited number of insecticides [2]. The World Health Organization Pesticide Evaluation Scheme (WHOPES) has mandated that ITNs only be treated with pyrethroid pesticides and IRS programmes use pyrethroids, a handful of organophosphates, carbamates, or DDT [3]. Resistance to this small arsenal of insecticides in vector populations poses a real threat to malaria control. Reports of African vector populations exhibiting resistance to insecticides began in the 1950s, and the problem continues to grow today [4]. In May 2012, WHO and partners issued the Global Plan for Insecticide Resistance Management in malaria vectors (GPIRM), to address this sustained growth in resistance [5]. This plan calls for redoubled efforts to manage public health pesticide use, and urges increased monitoring of insecticide resistance in endemic nations and further research into the causes of insecticide resistance.

Insecticide resistance occurs through physiological and behavioural changes on a population level that are set in motion by repeated environmental exposures to insecticides or other toxins, over time. Vectors can become exposed to insecticides used in agriculture from contamination of nearby breeding sites, and while it is difficult to delineate the effect of this early exposure in terms of adult resistance in vivo, vector larvae collected from sites with possible insecticide exposures and then reared in laboratory conditions can express resistance to insecticides as adults [6–17]. Target site resistance, caused by alteration of the molecular target of the insecticide [18], and metabolic resistance, whereby mosquitoes more quickly detoxify or eliminate insecticides [19], are the best-studied and most readily quantifiable mechanisms of insecticide resistance. Resistance may also develop by alterations in insecticide absorption at the point of cuticular contact, or by changes in mosquito behaviour.

A major source of insecticide exposure for malaria vectors is public health insecticide use, which is the main focus of the recent GPIRM. However, some of the earliest reports of insecticide resistance in Africa observed that agricultural insecticide use might have contributed to the selective pressure on anopheline mosquitoes [20–23]. Indeed, substantial agricultural developments following World War II often coincided with major malaria vector control campaigns [24]. Many of the pesticides used in agriculture overlap with those relied on by public health agencies, further complicating insecticide resistance management.

After the early suggestions of a connection between agricultural insecticide use and insecticide resistance in malaria vectors, researchers sought an analytic framework for understanding how and when a specific association might be inferred. In 1988, Lines described five types of evidence that could indicate a relationship [25]. The first two types of evidence he described are temporal, where regional resistance in a mosquito population either pre-dates public health insecticide use or varies with agricultural spraying seasons in that area. Spatial relationships make up another category, where observed resistance is higher in areas with agricultural insecticide use than in comparable non-agricultural areas. Another type of evidence arises if vector mosquitoes express resistance to the same insecticides used in agriculture: a chemical correlation. Finally, decreases in mosquito population size during agricultural spray seasons may also indicate selective pressure from agriculture. Following this initial phase of research, and some international acknowledgement of agriculture’s potential role in insecticide resistance, relatively few studies explored the relationship between agriculture and insecticide resistance further, though reports of newly resistant vector populations in Africa neither slowed nor diminished in urgency.

This period of relative silence in the scientific community ended with the publication of a landmark study in 2002 by Diabaté et al. [9]. Since then, research on the issue has increased considerably, supported by methodological improvements and increased funding for vector surveillance, and is providing important information to facilitate progress in insecticide resistance control. The evidence in this new work can help agencies now struggling to curb increasing insecticide resistance with stewardship policies that focus on public health insecticide use alone. Using a slight modification of the framework established by Lines [25], this review assembles and examines the recent research results, to evaluate the relationship between agricultural insecticide use and insecticide resistance in malaria vectors in Africa. In addition to assessing this relationship, other factors that may modify this interaction are identified, to provide a larger picture of the current problem and recommend future directions for the stewardship of key insecticides.

## Methods

To describe the body of evidence linking agricultural insecticide use with insecticide resistance in Africa, a revised set of criteria, shown in Table 1, was generated based on Lines’ five original evidence types. Lines’ evidence type based on observed declines in mosquito populations during agricultural sprayings was not included here due to infrequent reporting of appropriate data. An additional criterion was added based on reports that describe correlations between quantities of agricultural insecticides used and trends in insecticide resistance (‘correlation in quantity’). The revised criteria indicating agricultural-use selection for insecticide resistance are given in Table 1.

**Table 1:** Criteria for associating insecticide resistance in malaria vectors with agricultural insecticide use

Criterion	Example
*Timeline*: resistance in mosquitoes to a particular insecticide pre-dates public health or personal use of that insecticide at that site	Mosquito populations from Khartoum express 60–80 % mortality when exposed to the carbamate beniocarb, but carbamates are not approved for public health use in Khartoum, and are the most commonly used class of pesticides for agriculture [28]
*Correlation in time:* vector resistance levels rise and fall with the agricultural spraying schedule at a sampling site over time	Mosquitoes collected from cotton farms in Cameroon during the cotton spraying season showed a 1.6-fold increase in median knockdown time relative to mosquitoes collected from the same sites just prior to the spraying [18]
*Correlation in space*: vector populations from agricultural sites are more resistant to insecticides than their counterparts at non-agricultural sites in the same region	In Mali, the frequency of the *kdr* resistance gene was highest in mosquitoes sampled from a cotton-growing site, compared to sites with just personal or public health insecticide use history [30]
*Correlation in quantity*: vector resistance levels in a population increase with increasing quantity of agricultural insecticides in that region	Mosquito populations collected from two vegetable farms in Benin showed mortality to insecticides that varied inversely with concentrations of insecticides in the soil and water from these sites [25]
*Chemical correlation*: the insecticides to which a mosquito population is resistant correspond to the insecticides used for agricultural purposes in that area	At a site in Cameroon where pyrethroids make up a higher proportion of pesticides used in agriculture, mosquitoes showed higher pyrethroid resistance compared to mosquitoes from another agricultural site with less reliance on pyrethroids [26]

**Fig. 1:**
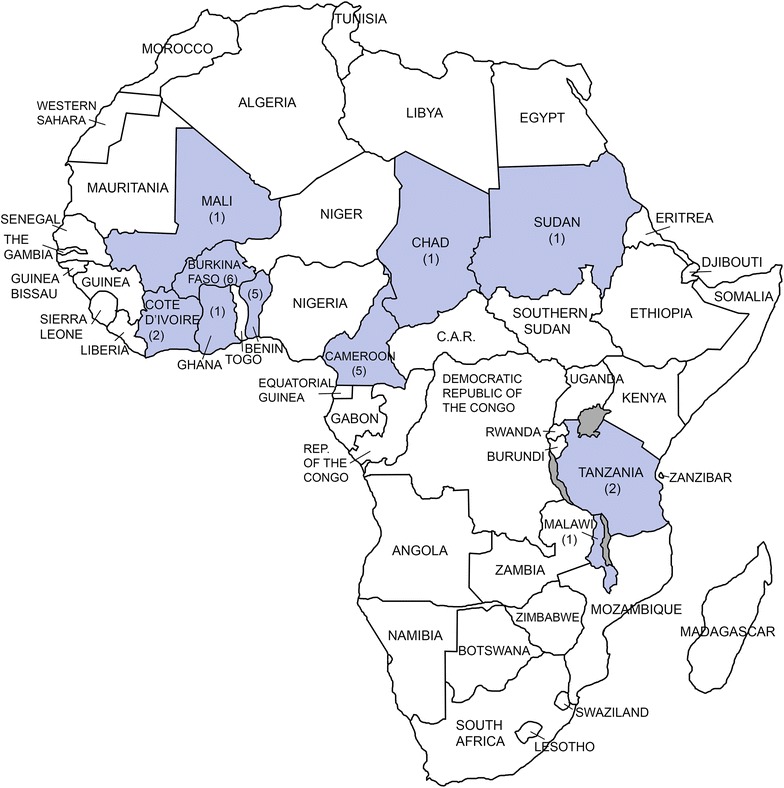
Map of study locations considered in this review. *Parentheses enclose number* of publications per county

Using Web of Science, WorldCat and PubMed search engines, literature related to agricultural insecticide use and insecticide resistance in malaria vectors was retrieved and reviewed. The primary search term was *impact of agriculture on insecticide resistance in malaria vectors Africa,* and the following additional terms were included for more directed inquiries: *Anopheles, mosquito, environment, crop, urban, peri*-*urban, rural, carbamates, DDT, pyrethroid, organophosphate, organochlorine,* or specific country names. Additional articles were located through paper references and cited-by lists, and by searching by author name. Peer-reviewed articles published in English or French since 2000 were considered. Twenty-five papers were found to directly assess the relationship between agriculture and insecticide resistance in African countries. A map of study locations by country for the 25 papers is shown in Fig. 1. The findings presented in these works were analysed with respect to the five criteria in Table 1, the simplified results of which are presented in Table 2.

**Table 2:** Reviewed publications by first author and year of publication

Paper	Country	Species	Resistant to	Timeline	Correlation in space	Correlation in time	Correlation in quantity	Chemical correlation
Diabaté et al. [9]	Burkina Faso	1	DDT, PYs		+	+		
Akogbéto et al. [33]	Benin	1	NA		+		+	
Tia et al. [15]	Cameroon	2	DDT, PYs		+		+	**+**
Corbel et al. [36]	Benin	1, 5	DDT, PYs		+			**+**
Tripet et al. [16]	Mali	2	DDT, PYs	+	+			**+**
Chouaïbo et al. [7]	Cameroon	1	DDT, PYs		+	+		**+**
Djouaka et al. [43]	Benin	2	PYs		+			**+**
Djogbénou et al. [10]	Burkina Faso	2	OPs, CMs		+			
Kerah-Hinzoumbe et al. [28]	Chad	1	PYs		+			
Klinkenberg, et al. [26]	Ghana	1	PYs		0			
Müller et al. [11]	Cameroon	3	PYs			+		**+**
Mzilahowa et al. [12]	Malawi	1, 4	DDT	+	+			**+**
Dabiré et al. [8]	Burkina Faso	1	DDT, PYs		+		+	
Dabiré et al. [29]	Burkina Faso	2	OPs, CMs		+		+	
Nwane et al. [34]	Cameroon	2	DDT, PYs		+	0	+	**+**
Antonio-Nkondjio et al. [44]	Cameroon	2	CMs, DDT, PYs		+			**+**
Djègbè et al. [31]	Benin	1	DDT, PYs		+			
Yadouléton et al. [17]	Benin	1	DDT, PYs			+	+	**+**
Badolo et al. [45]	Burkina Faso	1	DDT, PYs	+	+			**+**
Dabiré et al. [30]	Burkina Faso	1	OPs, CMs	+	+		+	
Abuelmaali et al. [35]	Sudan	3	OPs, CMs, PYs	+		0	+	**+**
Koffi et al. [27]	Cote d’Ivoire	1	OCs, CMs, PYs		+			
Nwane et al. [14]	Cameroon	1	DDT, PYs		0			
Nkya et al. [13]	Tanzania	1	PYs		+		+	
Nkya et al. [6]	Tanzania	2	OCs, OPs, PYs				+	**+**

+, tested and/or observed with positive results; 0, tested and/or observed with null or inconclusive results; Species 1, *Anopheles gambiae s.l.*; 2, *Anopheles gambiae s.s.;* 3, *Anopheles arabiensis;* 4, *Anopheles quadriannulatus*; 5, *Culex quinquefasciatus*. Plus marks indicate positive associations found by evidence type. *DDT* dichlorodiphenyltrichloroethane, *PYs* pyrethroids, *OPs* organophosphates, *OCs* organochlorines, and *CMs* carbamates

A number of different techniques to assess resistance, and terms to describe it were employed in this body of literature. Here, the terms resistance or resistance level refer to any measures of resistance, including per cent mortality, frequency of resistance alleles, or knockdown time. Resistance levels reported in different papers were not compared directly except in a crude analysis using the control sites of various papers as a random sample for comparison against certain test sites. Additional analyses were conducted to explore how associations between agricultural insecticide use and insecticide resistance in malaria vectors may be impacted by other variables. If in three or more papers the authors made mention of or measured a variable in addition to the agricultural status of their sampling locations, and that factor was associated with differences in observed resistance levels, it is discussed in this review.

## Results

### Evidence for agriculture contributing to insecticide resistance

Of the 25 papers evaluating the relationship between agricultural insecticide use and insecticide resistance, all but two papers [14, 26] described resistance scenarios that met one or more of the five criteria. Nineteen papers described evidence that met two or more criteria. The most frequently met criteria in these papers were correlation in space (19) and chemical correlation (13); the least frequent were descriptions of resistance pre-dating public health insecticide use or correlation between resistance and agricultural season (5 and 4, respectively). Both of the papers finding no conclusive association between agricultural insecticide use and vector resistance considered sites with potentially high insecticide exposures from non-agricultural uses, which may have confounded the results relevant to this review [14, 26]. The 25 papers are from study sites across malaria-endemic Africa (Fig. 1), confirming that the putative relationship between agricultural insecticide use and insecticide resistance in malaria vectors is widespread.

### Modifiers

Three additional social and behavioural factors emerged as potential modifiers of the relationship between agricultural insecticide use and insecticide resistance in vectors: crop type, pest management strategy and urban development. Each was discussed or quantified in three or more papers, and found to be associated with differences in population resistance levels.

### Crop type

Crop type was among the first factors recognized as modifying the relationship between agricultural insecticide use and insecticide resistance in malaria vectors. Diabaté et al. observed increased resistance at cotton growing sites, a finding subsequently supported in eight other papers from five different African countries [7, 8, 11, 17, 27–31]. This reported correlation between cotton and higher insecticide resistance mirrors cotton pesticide use data in Africa, indicating cotton as the cash crop with the highest intensity insecticide use of any crop [32].

In eight studies, vegetable cultivation strongly related to insecticide-resistant field collections [15, 16, 18, 27, 31, 33–36]. These results again correspond with pesticide use statistics for vegetable crops. Vegetable production requires significantly higher quantities and/or more frequent application of pesticides than other food crops [32, 37]. Vegetable cropping is also linked to increasing urban agriculture, and this intersection of factors may complicate or exacerbate the effects of vegetable cultivation practices on insecticide resistance in vectors. The impact of urban development is discussed below.

Irrigated crop production, especially rice, has increased in recent decades in Africa, and has raised concern over its potential impact on malaria vector control [38]. Seven of the studies reviewed here examined the insecticide susceptibility of vector populations at rice-growing sites [9, 10, 27, 28, 31, 36, 39], and found low-to-moderate resistance levels in these mosquito populations. In two of these studies, the moderate vector resistance at the rice site was attributed to cross-contamination from nearby cotton farms [9, 10]. While there is little generalizable data regarding insecticide use on rice crops in Africa, a recent survey conducted in Benin reported that insecticides were used in rice cultivation in three out of five districts, though quantity and frequency of application was not reported [40]. In addition, a study conducted in Cote d’Ivoire found very high levels of insecticide resistance in Anopheles mosquitoes collected from rice fields, though this report did not compare these resistance levels to a non-rice control [41]. This case of high resistance suggests the possibility that, in some instances, insecticide use on rice may play a larger role in the development of insecticide resistance than the seven papers reported here depict, but further research is necessary.

Additional crop types were included in this body of literature, but without sufficient overlap to posit any consistent pattern between them and insecticide resistance relative to other crops. Studies that included subsistence agriculture sites with no pesticide use found low resistance in vectors sampled from these locations [10, 31]. Overall, crop-based variation in resistance was found for cotton and vegetables, and insecticide use in rice cropping did not relate to resistance.

### Pesticide use and pest management strategy

Farmers and agricultural operation owners determine insecticide type, quantity and application frequency depending on costs, government and industry recommendations, producer instructions, community precedents, and personal experience [42]. Three of the studies reviewed here conducted surveys of agricultural workers and farm owners along with field sampling at the same sites [17, 34, 35]. Abuelmaali et al. found increased insecticide resistance at locations where farm workers reported that they applied pesticides more frequently, were more likely to switch pesticide class if inefficacy was perceived, and were less likely to properly dispose of unused or expired pesticides, compared to other sites. Nwane et al. found that mosquitoes collected from an agricultural area that reported a higher proportion of organophosphate and pyrethroid use had higher levels of resistance to those agents than mosquitoes from the comparison site. Yadouléton et al. found vector population resistance levels that correlated directly with reported insecticide use in three different pest management strategies. Thus, the three studies that collected simultaneous site-specific data both on agricultural insecticide use as practised and on resistance in vector populations indicated an association between pesticide usage behaviour for agricultural purposes and insecticide resistance in malaria vectors.

### Urban development

Metabolic resistance in vectors may develop from exposures beyond insecticides, including urban pollution or other environmental toxins. Urban centres characteristically have higher levels of pollution, and until recently, this, in combination with personal insecticide use for nuisance abatement, constituted the extent of vector exposures to toxins in cities; any insecticide resistance observed was attributed to these sources [4]. The recent development of urban farming in Africa has added a new dimension to the urban environment, and a complex modifier of relationships between agricultural insecticide use and insecticide resistance in malaria vectors.

The first comparison made possible by the papers reviewed here concerns differences in resistance between urban centres with city farming and those without. A crude appraisal of mosquito populations from cities with and without agriculture suggests that a higher proportion of agricultural cities have resistant mosquito populations:

- Of the ten papers reporting on cities with no record of urban agriculture: five found moderate-to-high [9, 10, 13, 43, 44] and five found low-to-non-existent [6, 7, 33, 34, 45] levels of insecticide resistance;
- Of the 12 papers reporting on cities with noted urban agriculture activity: nine found moderate-to-high [15, 27, 28, 31, 34–36, 44, 45] and three found mixed moderate-to-high and low-to-non-existent [8, 29, 30] levels of insecticide resistance.

These last three papers were all based on the same location and conducted by the same research group. Four papers sampled at *both* agricultural and non-agricultural urban sites simultaneously [26, 34, 44, 45], thus controlling for region, season, and research team. Three out of four of these papers reported higher resistance levels in vector mosquito populations from the agricultural urban sites than from sites in cities without farming [34, 44, 45]. These three papers, and the crude comparison, suggest that urban agricultural land use may foster increased insecticide resistance in malaria vectors.

The second comparison is between vector populations in rural farming areas and those in urban farming areas. Nine papers sampled at both rural and urban agricultural sites: six groups found higher levels of resistance in urban than in rural agricultural sites [8, 15, 29, 30, 36, 45, 46], two found split results [27, 28], and one found lower resistance in urban mosquito populations than in rural ones [34]. The one study that matched their urban and rural farming sites by crop type found similar resistance levels in these pairs [27]. These results tentatively suggest that agriculture in urban settings may exacerbate insecticide resistance in vector populations, but potential crop-type interactions and somewhat mixed results temper this association.

These studies overall suggest that cities with urban farming may be associated with higher vector resistance than non-agricultural cities, and potentially higher resistance than rural agricultural sites. Possible relationships between peri-urban agriculture and vector insecticide resistance remain largely unexplored [46].

## Discussion

The recent revival in research on agriculture’s putative role in the development of insecticide resistance in African malaria vectors has created an opportunity to re-evaluate current strategies for managing insecticide resistance and reconsider the best directions for policy. This review presents an updated set of criteria, and several key modifying factors, by which possible associations between insecticide use in agriculture and the development of insecticide resistance may be examined, and uses these to evaluate the 25 relevant recent publications. In 23 of the 25, from across Africa, higher resistance in mosquito populations was associated with agricultural insecticide use. Crop type had a strong impact on the extent of insecticide resistance associated with agricultural insecticide use: cotton was most commonly associated with higher resistance in mosquito populations, followed by vegetables, while rice was not associated with resistance. Farm operators’ pest management strategies for insecticide use were associated with insecticide resistance patterns in vector populations. Last, agriculture in urban areas was associated with higher levels of vector resistance than found in urban areas without farming.

Among the limitations of this broad review are that, by necessity, all types of insecticides and all forms of resistance were grouped together. Articles published in languages other than English and French were not included. Relatively few of the African nations experiencing insecticide resistance among malaria vectors were represented in this literature. In addition to these limitations, it is difficult to predict the potential impact that agricultural insecticide use may have on malaria outcomes in humans [47]. While in general, high levels of insecticide resistance do associate with some degree of vector control failure, there are cases where vector control has remained effective despite higher resistance levels [48, 49]. Additionally, it is important to consider that this review is drawn from a collection of papers that are essentially snapshots of insecticide resistance at various points and places in time. Each individual study incorporated here is subject to its own limitations. This review should therefor not be taken as conclusive evidence for any larger conclusions, but rather as a summary of what evidence so far exists to help understand the potential role agricultural insecticide use may have in the development of insecticide resistance in African malaria vectors.

## Conclusions

Further research should seek to quantify the sources and scope of vector mosquito exposure to agricultural insecticides, ideally incorporating resistance surveillance systems already in place. A better understanding of insecticide use in practice and current pest management strategies is needed to estimate magnitudes of insecticide exposures from agriculture and prioritize insecticide resistance control efforts accordingly. The impact of urban agriculture in particular requires further exploration, with studies that take different crop types and peri-urban areas into consideration.

Translational research should aim to evaluate possible systems for the stewardship of important insecticides. Development of integrated pest management strategies that are applicable and economically feasible for specific affected regions is crucial, as is outreach and education for the myriad agricultural workers whose decisions impact the health of their communities as well as their own livelihoods. On a policy level, at any geographic scale, the responsibility for addressing insecticide failures cannot fall solely on public health agencies.

## Abbreviations

DDT: dichlorodiphenyltrichloroethane
PYs: pyrethroids
OPs: organophosphates
OCs: organochlorines
CMs: carbamates
IRS: indoor residual spraying
ITN: insecticide-treated net
